# Serum creatinine/cystatin C ratio as a surrogate marker for sarcopenia in patients with gastric cancer

**DOI:** 10.1186/s12876-022-02093-4

**Published:** 2022-01-19

**Authors:** Jing Sun, Hui Yang, Wentao Cai, Jingwei Zheng, Ningzhe Shen, Xinxin Yang, Bujian Pan, Weiteng Zhang, Xiaodong Chen, Xian Shen

**Affiliations:** 1 Department of Gastrointestinal Surgery, The Second Affiliated Hospital, Wenzhou Medical University, Wenzhou, Zhejiang China; 2 Department of Gastrointestinal Surgery, The First Affiliated Hospital, Wenzhou Medical University, Wenzhou, Zhejiang China

**Keywords:** Serum Cr/CysC ratio, Sarcopenia, Gastric cancer

## Abstract

**Background:**

Sarcopenia is an age-related syndrome that may have negative impact on surgical outcomes and long-term survival of patients with gastric cancer. Serum creatinine/cystatin C (Cr/CysC) ratio has attracted attention as a surrogate marker for sarcopenia but has not been adequately studied in patients with gastric cancer. The purpose of this study was to investigate the validity of serum Cr/CysC ratio as a predictor of sarcopenia, evaluate a statistical cut-off value, and assess the relationship between Cr/CysC ratio and prognosis of patients with gastric cancer.

**Methods:**

We retrospectively studied 327 patients who underwent surgery for gastric cancer from June 2009 to October 2021. The skeletal muscle mass index was calculated using computed tomography (CT). We determined the relevance of serum Cr/CysC ratio as a surrogate maker for sarcopenia by comparing it with various biomarkers. The Concordance index (C-index) was calculted to measure whether the Cr/CysC ratio can prognosis of patients with gastric cancer.

**Results:**

Serum Cr/CysC was significantly correlated with with Skeletal Muscle Index (SMI) (r = 0.221, p < 0.001) and Skeletal Muscle Area (SMA) (r = 0.258, p < 0.001). The area under the curve for sarcopenia was significantly larger for serum Cr/CysC ratio than for other biomarkers (Cr/CysC: 0.644, CysC: 0.535, Cr: 0.556). Patients in the high-Cr/CysC group have longer survival time than that in low-Cr/CysC group, defined by the cutoff value 0.67. The C-index of both Cr/CysC ratio and SMI with OS was 0.63.

**Conclusions:**

Serum Cr/CysC ratio can be used accurately, inexpensively, and easily to evaluate sarcopenia in male patients with gastric cancer. Our study shows that patients with Cr/CysC below 0.67 had possibility of sarcopenia and would be poor prognosis.

## Background

Gastric cancer is one of the most common malignant tumors worldwide. The incidence (5.7%) and mortality (8.2%) rates were fifth and third, respectively in 2018 [1]. Although development of technology has led to progress in tumor diagnosis, surgical methods, and adjuvant treatment, the survival rate of patients with gastric cancer remains low [2].

Sarcopenia is an age-related syndrome characterized by progressive and extensive loss of skeletal muscle mass and strength [3]; this has a negative impact on surgical outcomes and long-term survival of patients with gastric cancer [4]. Studies have shown that weight loss and malnutrition are common problems in patients with gastric cancer [5, 6].

In recent years, researchers have proposed a new method to predict muscle mass. Routinely measured serum creatinine (Cr) and serum cystatin C (Cys C) were used to assess renal function. Since the concentration of serum creatinine is affected by muscle mass, patients with decreased muscle mass will decrease serum creatinine [7, 8]; while serum Cys C is not affected by muscle mass [9, 10]. Based on the characteristics of these two, the method of dividing serum creatinine by serum Cys C can be used to further predict muscle mass. This method has been confirmed in the research of many diseases [11–14]. However, there are no studies to test the effectiveness of Cr/Cys C in patients with gastric cancer. Based on the above reasons, we are thinking about whether we can explore the prediction of Cr/Cys C for muscle mass in gastric cancer patients, and determine the best cut-off value of Cr/CysC ratio for predicting sarcopenia and its impact on the survival time of gastric cancer patients.

## Materials and methods

### Patients and study design

We obtained patient data from the Second Affiliated Hospital and Yuying Children’s Hospital of Wenzhou Medical University from June 2009 to May 2021. Inclusion criteria were as follows: (a) pathological diagnosis of gastric adenocarcinoma, (b) underwent radical gastrectomy, (c) underwent computed tomography (CT) imaging within 1 month preoperatively, and measure the concentration of serum creatinine and serum cystatin C whitin 1 week preoperatively. The exclusion criteria were as follows: (a) a diagnosis of metastatic or remnant gastric cancer, (b) received preoperative chemotherapy or radiotherapy, (c) having incomplete or inaccurate medical records, and (d) renal function impairment (an estimated glomerular filtration rate of < 60 mL/min/1.73 m^2^). After applying these criteria, 327 patients were recruited. In this study, the treatment of gastric cancer was developed according to the 2010 Japanese Gastric Cancer Treatment Guidelines [5]. Each patient provided their written informed consent to participate in this study. The research protocol was approved by the ethics committee. This research was conducted in accordance with the principles of the Declaration of Helsinki.

### Data collection

We collected the following clinical information from each patient: (1) preoperative personal information, including age, sex, height, American Society of Anesthesiologists (ASA) grade, complications, and preoperative serum creatinine and serum cystatin C; (2) postoperative conditions, including tumor location, tumor size, tumor histology type (tumor differentiation divided into high, medium, and low), pTNM staging, and chemotherapy. Tumor staging conforms to the 8th edition of the corresponding American Joint Committee on Cancer guidelines [15]. To reduce bias, all evaluations were performed by two independent, well-trained researchers and all other data were ignored.

### Definition of sarcopenia

Abdominal CT scan is routinely performed before surgery. Skeletal muscle mass was estimated by calculating the area of all skeletal muscles on the cross-section of the third lumbar vertebra (L3) [16] with ImageJ software. Tissues with a specific Hounsfield unit threshold of -29–150 were considered as skeletal muscles and were standardized according to height (m^2^), and the skeletal muscle mass was evaluated by calculating the skeletal muscle mass index (SMI) (cm^2^/m^2^) [17].

The criteria for sarcopenia were low skeletal muscle mass, low muscle strength, and/or poor physical function according to the European Working Group on Sarcopenia in Older People (EWGSOP) [18] and the Asian Working Group for Sarcopenia (AWGS) [19]. We used the following cut-off values to define sarcopenia: (1) SMI (L3) < 40.8 cm^2^/m^2^ for men and SMI < 34.9 cm2/m2 for women, considering possible racial differences indicating low muscle mass [20]. (2) Hand grip strength < 28 kg among men and < 18 kg among women were regarded as low muscle strength [19]; and (3) 6-m usual gait speed < 1.0 m/s was regarded as low physical performance [19].

### Follow-up

All patients were followed up in the outpatient department in the first month after surgery. After that, the patients were followed up by telephone or in an outpatient clinic every 36 months. The overall survival (OS) was defined as the time between surgery and death from any cause or the date of the last follow-up. We choose the patients from June 2009 to October 2017. All patients were followed up for at least 3 years. The last follow-up was in October 2020.

### Laboratory measurements

Serum Cr levels were measured at our hospital laboratory. Serum Cr levels were measured using an enzymatic method. Serum cystatin C concentrations were determined by using a particle-enhanced immunoturbidimetric assay, and Cr/Cys C ratio was calculated by diving serum Cr by serum Cys C.

### Statistical analysis

All continuous data in this study were non-normally distributed according to the Kolmogorov–Smirnov test. Therefore, continuous data were presented as median and interquartile range (IQR). Kaplan–Meier survival analysis were used to calculate the effects of Cr/CysC ratio on survival time. Receiver operating characteristic (ROC) curve analysis was used to evaluate the utility of the Cr/CysC ratio for identifying low SMI, based on the area under the ROC curve (AUC) and 95% confidence interval (CI). The Y ouden index (sensitivity + specificity-1) was calculated to determine the optimal cutoff points for the Cr/CysC ratio. The C-index was calculated to measure the impact of Cr/CysC ratio and SMI on prognosis. All P values were two-sided, and a P value of < 0.05 was used to denote statistical significance. All statistical analyses were performed using Statistical Package for the Social Sciences, version 26.0 (IBM Corp., Armonk, NY, USA) and R version 4.1.2 (The R Foundation, Vienna, Austria).

## Results

### Patient characteristics

**Fig. 1:**
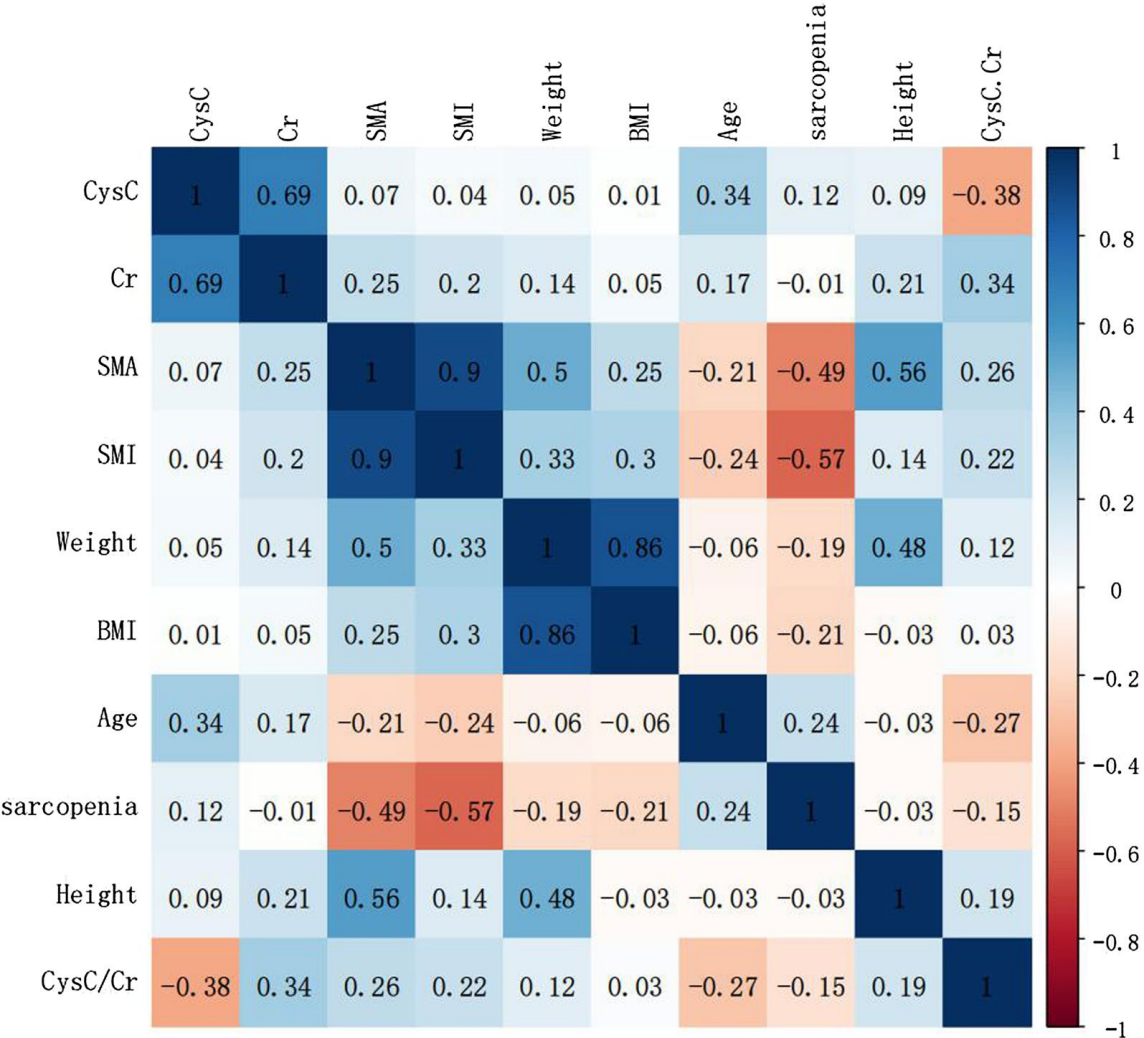
Correlation heat map analysis between factors which are basic characteristics of the population

We included 339 adults to participate in this study from June 2009 to October 2021. Some participants were excluded because of an estimated glomerular filtration rate of < 60 mL/min/1.73 m^2^ (n = 12). A total of 327 participants (median age: 64 years old, range 20–89 years old) were considered eligible for the analysis, including 234 men and 93 women, whose characteristics are shown in Table 1. There were 26 sarcopenia patients (11.1%) out of 234 men, and 16 sarcopenia patients (17.2%) out of 93 women among the patients. We conducted a correlation analysis on the basic characteristics of the sample. As shown in the heat map (Fig. 1), the serum CysC/Cr ratio is positively correlated with SMA, SMI, weight, height, and BMI, and negatively correlated with age and sarcopenia, but there is no strong correlation.

**Table 1:** Clinical characteristics of the male and female groups

Factors	Male (n = 234)	Female (n = 93)
Age (years)	63.3 ± 11.2	59.5 ± 12.8
Weight (kg)	61.9 ± 11.3	55.0 ± 8.5
Height (m)	1.68 ± 0.1	1.58 ± 0.1
BMI (kg/m^2^)	21.8 ± 3.7	22.0 ± 3.2
SMA (CT scan, kg)	139.3 ± 20.9	102.4 ± 18.2
SMI (kg/m^2^)	48.9 ± 7.2	40.9 ± 7.2
Serum cystatin C (mg/L)	1.0 ± 0.3	0.8 ± 0.2
Serum creatinine (mg/dL)	0.8 ± 0.2	0.6 ± 0.1
Serum Cr/Cys C	0.8 ± 0.2	0.7 ± 0.1
Sarcopenia, n%	11.1%	17.2%

### Correlation between clinical factors for sarcopenia

We performed Logistic regression analysis to evaluate whether Cr/Cys C is related to sarcopenia. Univariate analysis showed that Cr/Cys C, age, lower BMI, preoperative anemia, tumor size, and pTNM stage are predictors of sarcopenia. And Cr/Cys C (p = 0.01) is still an independent predictor of sarcopenia in multivariate analysis (Table 2). In addition, other markers including age ≥ 75 years, lower BMI have also been shown to be associated with increased risk of sarcopenia (all p < 0.05).

**Table 2:** Univariate and multivariate analyses the risk of sarcopenia

Factors	Odds ratio for sarcopenia (95% CI)			
	Univariate analysis	p value	Multivariate analysis	p value
Age				
> 75/ ≤ 75	8.543 (4.056–17.998)	< 0.001*	10.035 (4.217–23.877)	< 0.001*
Sex				
Male/female	1.662 (0.846–3.266)	0.140		
BMI, kg/m^2^				
≥ 18.5/< 18.5	0.177 (0.085–0.369)	< 0.001*	0.144 (0.062–0.337)	< 0.001*
Anemia				
Yes/no	2.091 (1.032–4.235)	0.041*	1.607 (0.705–3.666)	0.259
Tumor location				
Corpus/cardia	1.012 (0.388–2.641)	0.980		
Pylorus/cardia	1.020 (0.426–2.441)	0.965		
Mixed or total/cardia	0.614 (0.070–5.415)	0.660		
Tumor size				
≥ 4.75/< 4.75	2.100 (1.079–4.086)	0.029*	1.975 (0.842–4.632)	0.118
Type of differentiation				
Moderate/well	1.151 (0.376–3.528)	0.805		
Poor/well	0.955 (0.274–3.337)	0.943		
pTNM stage				
II/I	2.819 (1.046–7.595)	0.040*	2.639 (0.716–9.731)	0.145
III/I	2.409 (1.040–5.580)	0.040*	1.905 (0.595–6.599)	0.278
Serum Cr/Cys C				
Low/high	0.336 (0.163–0.692)	0.003*	0.334 (0.144–0.773)	0.010*

Asterisks indicate meaningful results

### Association between various biomarkers and sarcopenia

**Fig. 2:**
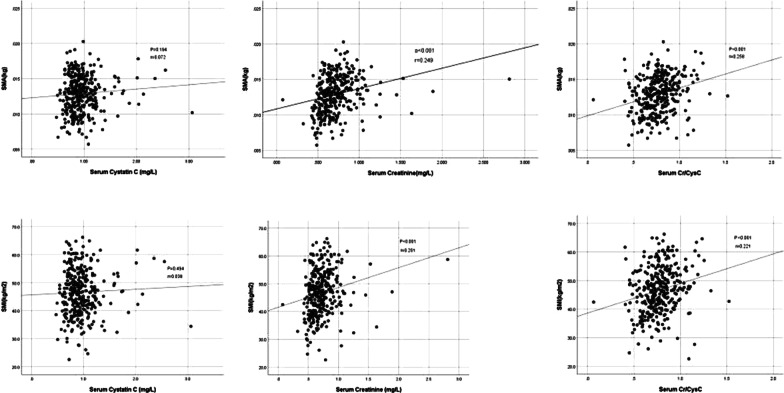
Linear correlation between the skeletal muscle mass index (SMI), skeletal muscle area (SMA) and cystatin C, creatinine and creatinine/cystatin C ratio in the group

**Fig. 3:**
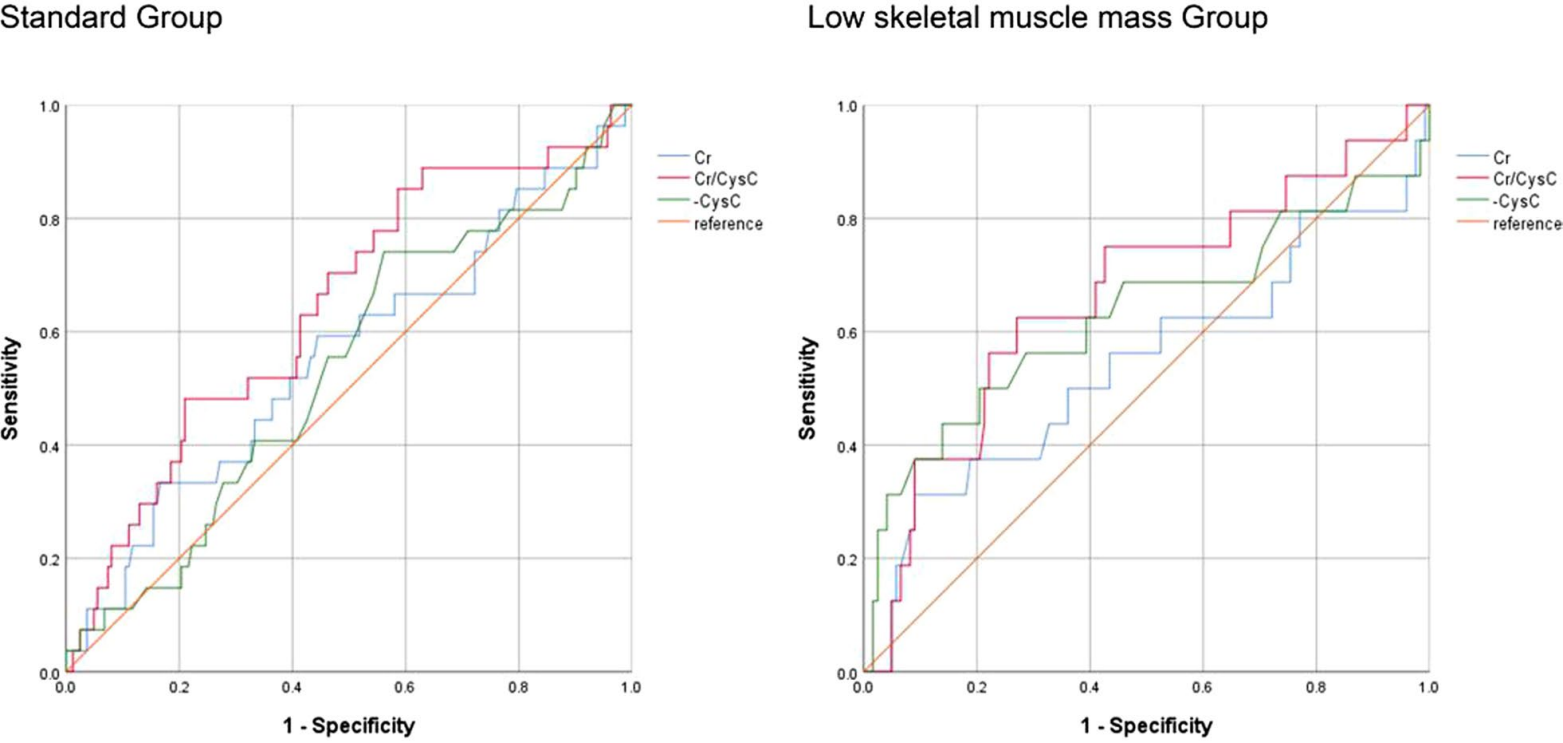
Receiver operator characteristic curves presenting sarcopenia and low skeletal muscle mass according to serum creatinine (Cr)/cystatin C (CysC) ratio, serum CysC and serum Cr levels

Serum Cr/CysC ratio was positively correlated with SMI (r = 0.221, p < 0.001) and SMA (r = 0.258, p < 0.001). Serum Cr was positively correlated with SMI (r = 0.201, p = 0.002) and SMA (r = 0.249, p < 0.001), while serum CysC had no significant correlation with SMI and SMA (p > 0.05) (Fig. 2). We calculated the AUC of each biomarker using the ROC curve (Fig. 3), and used the DeLong test to check the effectiveness of each biomarker as a predictor of sarcopenia. We divided the patients into two groups according to whether to measure hand grip strength and 6-m usual gait speed, a standard group that measured muscle mass and a low skeletal muscle mass group that only measured muscle area. Serum Cr/CysC ratio, CysC, Cr AUC were 0.664 (95% CI 0.511–0.816), 0.630 (95% CI 0.452–0.808), 0.543 (95% CI 0.367–0.719) in standard group. And Serum Cr/CysC ratio, CysC, Cr AUC were 0.644 (95% CI 0.532–0.757), 0.535 (95% CI 0.418–0.652), 0.556 (95% CI 0.432–0.681) in low skeletal muscle mass group. The AUC of the Cr/CysC ratio in two group were both significantly greater than the AUC of all other biomarkers (p = 0.034 and p = 0.016).

### Cutoff value of serum Cr/CysC on sarcopenia

**Fig. 4:**
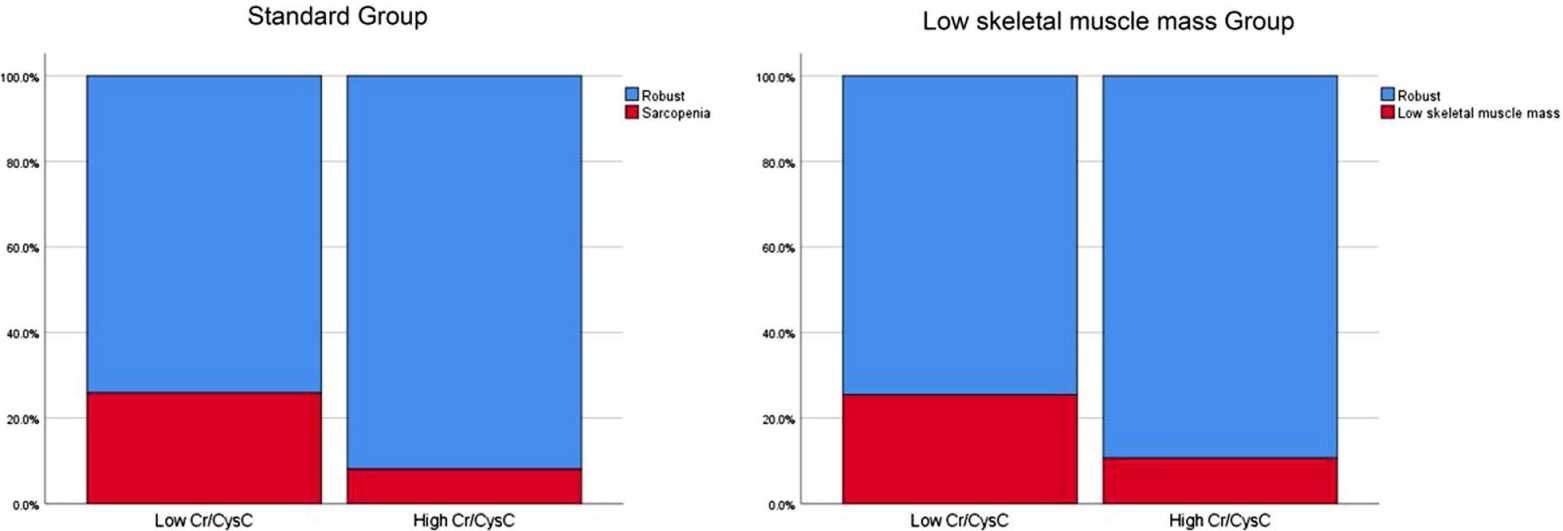
Frequency of sarcopenia and low skeletal muscle mass in patients with low and high serum creatine (Cre)/ cystatin C (Cys C) ratio. Cr/CysC < 0.67 was defined as low Cr/CysC, and Cr/CysC S 0.67 was defined as high Cr/CysC

We calculated the serum Cr/CysC cutoff to be 0.67, using Youden-Index that maximizes the value of “sensitivity þ specificity-1”. Based on the calculated cut-off value, in the Low skeletal muscle mass group, the sensitivity, specificity, positive predictive value, and negative predictive value were 0.26, 0.87, 0.25 and 0.88, respectively. And the sensitivity, specificity, positive predictive value, and negative predictive value were 0.44, 0.84, 0.26 and 0.92 in the standard group (Fig. 4).

### Survival outcomes

**Fig. 5:**
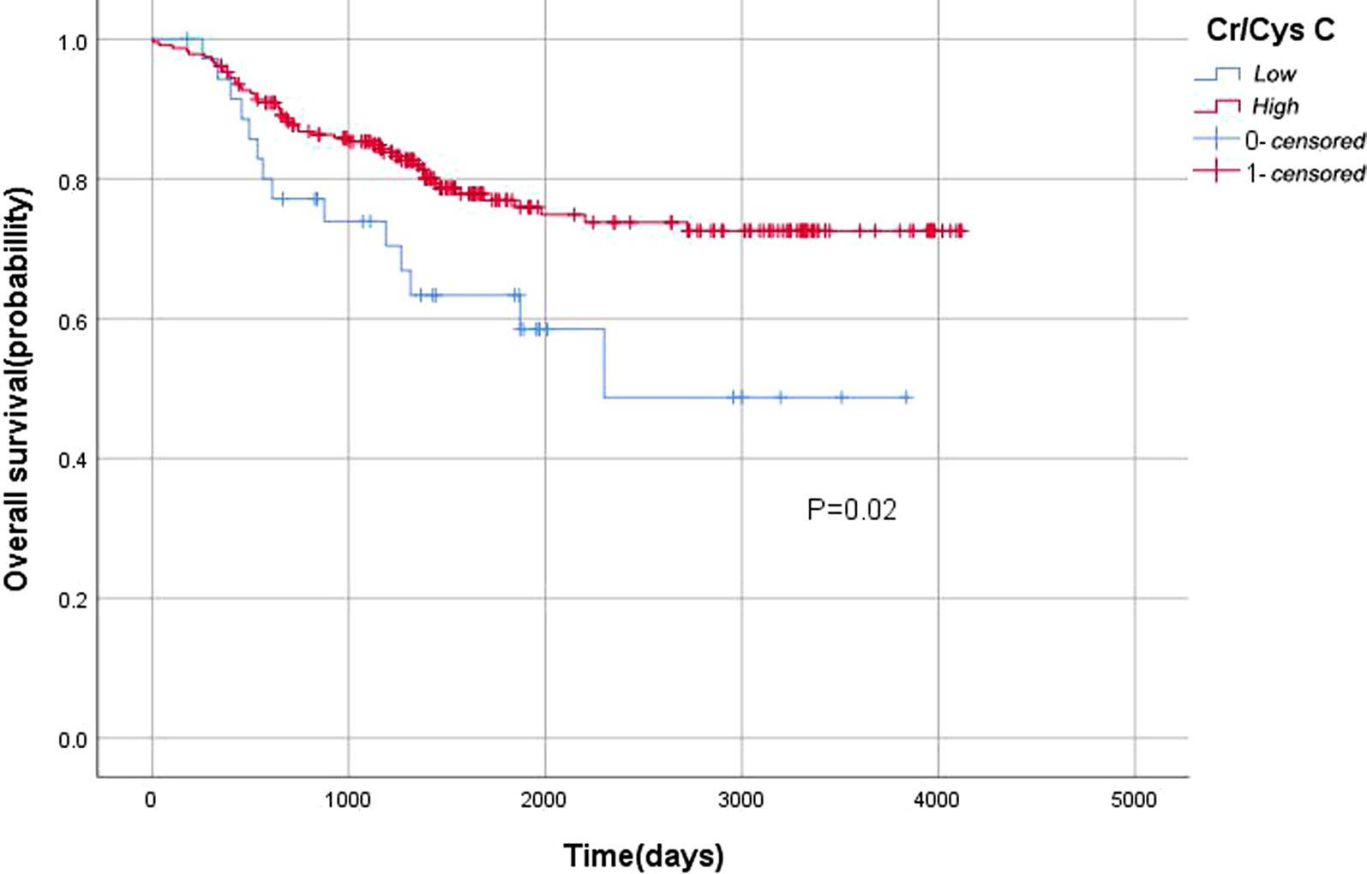
Kaplan–Meier survival analyses of patients in low and high serum creatine (Cre)/ cystatin C (Cys C) ratio. Cr/CysC < 0.67 was defined as low Cr/CysC, and Cr/CysC S 0.67 was defined as high Cr/CysC

We used the calculated optimal cut-off value to draw the KM curve of serum Cr/CysC and survival time after excluding 2 patients who died within 1 month after surgery in order to reduce the impact of postoperative acute complications on survival time. As shown in the Fig. 5, serum Cr/CysC is highly correlated with OS. The OS of the high serum Cr/CysC group was significantly longer than that of the low serum Cr/CysC group (p = 0.02). We used age, BMI, pTNM stage and tumor size to perform COX regression to perform survival multivariate analysis (Table 3). And Cr/Cys C (HR = 0.565 (95% CI 0.311–1.025); p = 0.060) is marginal significant, which needs expand the sample size and eliminate confounding factors for further analysis (Table 3). In the R software, we calculated the height-calibrated Cr/CysC ratio, SMI and survival C-index value. The C-index of SMI and OS was 0.62. And after calibrationed by height, the C-index of Cr/CysC ratio and OS was 0.56. When they both as variables, we calcualted the C-index as 0.63, which higher than that of SMI.

**Table 3:** Univariate and multivariate analyses of factors in relation to overall survival

	Univariate analysis		Multivariate analysis	
	HR (95% CI)	p value	HR (95% CI)	p value
Serum Cr/Cys C low/high	0.501 (0.277–0.906)	0.022*	0.565 (0.311–1.025)	0.060

We use age, BMI, pTNM stage and tumor size to perform COX regression to perform survival multivariate analysis

Asterisks indicate meaningful results

## Discussion

Baumgartner et al. first proposed the concept of "sarcopenia" [21] to describe the decrease in muscle mass of the elderly with age in 1998. The occurrence of sarcopenia is closely related to age and special physiological conditions which has received extensive attention from scientists in recent years. Many studies have shown that sarcopenia is associated with an increased risk of recurrence [22], shortened survival time [23, 24] and increased other causes of death [25] after gastric cancer resection. Weight loss and malnutrition are issues that are very worthy of our attention in all stages of gastric cancer treatment. More than half of patients with gastric cancer have some degree of weight loss due to the potential impact of the tumor at the time of diagnosis [26]. The inability to maintain weight is considered to be a poor prognostic factor that affects long-term survival during neoadjuvant therapy or chemotherapy [27, 28].

The relationship between Cr/CysC ratio and sarcopenia in different populations has been discussed. Tetsuka et al. found for the first time that the Cr/CysC ratio of patients with amyotrophic lateral sclerosis was lower than that of healthy people [29]. In the Japanese elderly without severe renal impairment, the Cr/CysC ratio is positively correlated with muscle mass and physical function [13]. The reduction of Cr/CysC ratio is considered to be a predictor of sarcopenia in patients with type 2 diabetes and COPD [11, 30]. Recent studies have found that the Cr/CysC ratio can also predict malnutrition, weakness and poor clinical outcomes in ICU patients [31–33]. Studies have also confirmed that this indicator can be used as an effective surrogate indicator for evaluating sarcopenia in patients after colorectal cancer surgery [34]. However, there is no report confirming the association between Cr/CysC ratio and sarcopenia in patients with gastric cancer.

This study shows that the serum Cr/CysC ratio is a useful predictor of sarcopenia compared with other biomarkers (such as serum Cr, CysC). In addition, the best cut-off value of serum Cr/CysC ratio is 0.67. Patients with Cr/CysC ≥ 0.67 can basically rule out sarcopenia.

We found that the Cr/CysC ratio is the most predictive of sarcopenia in patients with gastric cancer which is positively correlated with SMI and SMA among the three biomarkers of serum Cr, serum CysC and serum Cr/CysC ratio in this study. Previous studies have shown that serum Cr is related to muscle mass [35, 36]. In our study, serum Cr is positively correlated with SMI and SMA which is consistent with the results of previous studies. Serum CysC does not show any correlation with muscle mass. As an indicator of renal function, serum CysC has received more and more attention in recent years. It is a low molecular weight protein with a stable production rate and can be freely filtered by the glomerulus [19]. Therefore, the Cr/CysC ratio obtained by calibrating Cr with CysC, that is not affected by muscle mass, can predict sarcopenia.

We found that the best cut-off value of the optimal serum Cr/CysC ratio for predicting sarcopenia in patients with gastric cancer is 0.67. A study found that the optimal cut-off value of Cr/CysC ratio in diabetic patients for predicting sarcopenia was 0.9 [11], while another study found that the optimal cut-off value of Cr/CysC ratio was 0.71 in COPD patients [30]. However, there is no study to detect the optimal cut-off value of Cr/CysC ratio for sarcopenia in gastric cancer patients as we know.

The OS of the high serum Cr/CysC group defined by the optimal cut-off value was significantly higher than that of the low serum Cr/CysC group. And sarcopenia shorted the survival time of patients as an independent factor affecting the prognosis of gastric cancer patients which result is consistent with that of Cr/CysC ratio. We also constructed height-calibrated Cr/CysC ratio and SMI and survival models. Studies have shown that although the Cr/CysC ratio is related to OS, the correlation is not as good as SMI. But its correlation is greater than SMI when it is used together with SMI as an indicator to measure the prognosis of patients with gastric cancer. This means that the prognosis of gastric cancer patients can be predicted by using the Cr/CysC ratio and SMI as a combined index.

In this study, we used the area of all skeletal muscle on the CT scan at the L3 level as the standard for estimating the patient's skeletal muscle mass. Many studies have confirmed that CT scans are an effective way to assess body composition and can predict sarcopenia in the population. It has been widely used in oncology as a highly feasible method. However, it is still subject to many limitations in actual clinical applications as a method of evaluating body composition, such as its high radioactivity and high cost. In addition, the measurement of grip strength and pace is often used to assess sarcopenia. But this method is very dependent on patient compliance and is often difficult to implement in clinical operations. However, early diagnosis and intervention are very important since sarcopenia can significantly affect the prognosis of patients with gastric cancer. Serum Cr/CysC ratio is a simple, easy and low-cost method that can initially screen patients for sarcopenia and assist subsequent treatment and intervention. If the Cr/CysC ratio is lower than 0.67, detailed examinations must be carried out by BIA, DEXA, CT or MRI.

This study also has certain limitations. First of all, it is a retrospective study and sometimes there are certain deficiencies when collecting follow-up information. Second, the sample size is insufficient because this study only included data from a tertiary center for statistical analysis. The detection of serum Cr and serum CysC will also be affected by reagents and instruments. In follow-up studies, prospective studies can be carried out around this point of view and verified in other centers. Third, this study only referred to the Chinese population. Due to the different criteria for sarcopenia, this limits the applicability of the conclusions, especially for Western populations. In follow-up research, how to determine the appropriate standards according to different groups of people to verify our conclusions is very important.

## Data Availability

The data that support the fndings of this study are available from the corresponding author upon reasonable request. Emails could be sent to the address below to obtain the shared data: shenxian@wmu.edu.cn.

## Abbreviations

Cr/CysC: Serum creatinine/cystatin C
CT: Computed tomography
ASA: American Society of Anesthesiologists
SMI: Skeletal muscle mass index
EWGSOP: European Working Group on Sarcopenia in Older People
AWGS: Asian Working Group for Sarcopenia
OS: Overall survival
IQR: Interquartile range
ROC: Receiver operating characteristic
AUC: Area under the ROC curve
CI: Confidence interval
COPD: Chronic obstructive pulmonary disease
ICU: Intensive care unit
MRI: Magnetic resonance imaging
